# Estimation of Salt Intake Assessed by 24-Hour Urinary Sodium Excretion among Somali Adults in Oslo, Norway

**DOI:** 10.3390/nu10070900

**Published:** 2018-07-13

**Authors:** Sairah L. Chen, Cecilie Dahl, Haakon E. Meyer, Ahmed A. Madar

**Affiliations:** 1 Department of Community Medicine and Global Health, Institute of Health and Society, University of Oslo, 0315 Oslo, Norway; s.l.f.chen@studmed.uio.no (S.L.C.); cecilie.dahl@medisin.uio.no (C.D.); h.e.meyer@medisin.uio.no (H.E.M.); 2 Division of Mental and Physical Health, Norwegian Institute of Public Health, 0403 Oslo, Norway

**Keywords:** salt, urinary sodium and potassium excretion, Somali immigrants, 24-hour urine collection, Norway

## Abstract

High dietary salt intake is associated with increased blood pressure (BP) and cardiovascular disease (CVD) risk. The migration of Somalis from East Africa to Norway may have altered their dietary habits, making them vulnerable to adverse health outcomes. Since little is known about the lifestyle and health status of this population, the purpose of our study was to estimate salt intake in Somali adults in Oslo, Norway. In this cross-sectional study, we included 161 Somali adults (76 men, 86 women) from the Sagene borough in Oslo, Norway. Sodium and potassium excretion was assessed through the collection of 24-hour urine. Creatinine-based exclusions were made to ensure completeness of urine collections. Sodium excretion corresponding to an estimated dietary salt intake of 8.66 ± 3.33 g/24 h was found in men and 7.39 ± 3.64 g/24 h in women (*p* = 0.013). An estimated 72% of participants consumed >5 g salt/day. The Na:K ratio was 2.5 ± 1.2 in men and 2.4 ± 1.1 in women (*p* = 0.665). In conclusion, estimated salt intake was, while above the WHO recommendation, within the lower range of estimated salt intakes globally and in Western Europe. Further research is required to assess the health benefits of sodium reduction in this Somali immigrant population.

## 1. Introduction

Increased dietary salt intake is widely acknowledged as a major determinant for raised blood pressure and hypertension [1,2,3] and has been found to be associated with cardiovascular disease (CVD) outcomes [4,5]. According to the Global Burden of Disease Study 2015, hypertension has been the greatest contributor to mortality and morbidity over the last 25 years [6]. It is a leading risk factor for cardiovascular disease (CVD), which is responsible for one-third of all global deaths [7].

Globally, it is estimated that individuals consume between six and twelve grams of salt daily [8], far exceeding the WHO’s salt recommendation of five grams maximum [9]. However, baseline data on salt intake are limited, especially among low- and middle-income communities, including immigrant populations living in high-income countries. This is particularly troublesome considering growing evidence that immigrant communities bear a disproportionate burden of CVD. Estimating salt intake from dietary surveys is often unreliable [10]. The ‘gold standard’ method for estimating salt intake is 24-hour urine collection, as around 90% of ingested sodium is excreted in the urine [11,12,13]. However, the method is burdensome for researchers to conduct and participants to adhere to, often rendering 24-hour urine collection unfeasible [13].

There is little information about salt intake in the Norwegian population. The Somali community is Norway’s largest non-European immigrant group and there is currently no knowledge about salt intake in this population. This study, commissioned by the Norwegian Institute of Public Health, aims to estimate dietary salt intake among Somali immigrants in Norway by the use of 24-hour urinary sodium excretion. The baseline measurement is important for informing effective health promotion and for monitoring potential changes over time to meet targets in the global effort to prevent hypertension and cardiovascular events.

## 2. Methods

### 2.1. Population and Recruitment

This cross-sectional study design attempted to recruit all Somali adults (20–67 years) living in the *Sagene* borough of Oslo, Norway. The baseline survey was conducted between December 2015 and October 2016. Participants were recruited through a variety of community-based methods, including information meetings at activity centers, and through local Somali radio. Collaboration was established with local Somali organizations, a healthy life center, and three project assistants, identified by these partners, were trained to assist with recruitment and data collection. Door-to-door visits were conducted in order to access every known-Somali adult in the borough.

Conventional random sampling was deemed impractical as our previous experiences in recruiting immigrant populations through Statistics Norway resulted in low response rates [14]. We thus decided to limit the study to one borough in Oslo with a high population of people of Somali origin. Cultural sensitivity during the recruitment process was essential to increase the chance of participation. To establish trust between project leaders and potential participants, oral communication was preferred.

### 2.2. Urine Collection

Each participant was given a urine collection kit, comprised of a 3.0-L urine container with an integrated unit for closed urine transfer using the vacuum system, V-Monovette^®^. Instruction for use was given orally and in writing. The first morning void was to be discarded, but voids throughout the rest of the day until and including the morning void of the day after were instructed to be collected. Start time, end time, and irregularities were to be noted and the container kept cool throughout collection. Urine collection containers were returned, contents mixed, and total volume was recorded. Then, 2-mL samples of each participant’s urine containers were extracted and stored at −20 °C. All 2-mL samples were sent to the Medical Laboratory, University Hospital of North Norway (UNN) in one batch for one-kit analysis of urine volume, creatinine, sodium, and potassium levels. Urinary sodium and potassium were assessed using Roche Hitachi—an indirect ion-selective electrode to determine ion concentration [15]. The Medical Laboratory at UNN was accredited by Norwegian Accreditation according to the standard *NS-EN ISO 15189 TEST 209*.

### 2.3. Collection of Other Clinical and Demographic Factors

Blood pressure was measured using the Omron HBP 1300—a validated oscillometric device [16]. Participants were given a seated rest period of five minutes, and then blood pressure was measured three times consecutively for one-minute intervals. Blood pressure was estimated as the average of the second and third measurements. Hypertension was defined as >140 mmHg systolic and/or >90 mmHg diastolic and/or as being on antihypertensive treatment.

All participants were administered a questionnaire, employing the WHO STEPwise approach to chronic disease risk factor Surveillance (STEPS) [17]. Project assistants were responsible for helping participants complete the questionnaire on paper to ensure standard comprehension and response. Gender, age, number of years lived in Norway, and education level (years completed) were considered relevant variables in the current analyses as assessed by directed acyclic graph (DAG) analysis and literature review.

### 2.4. Sample Flow

**Figure 1:**
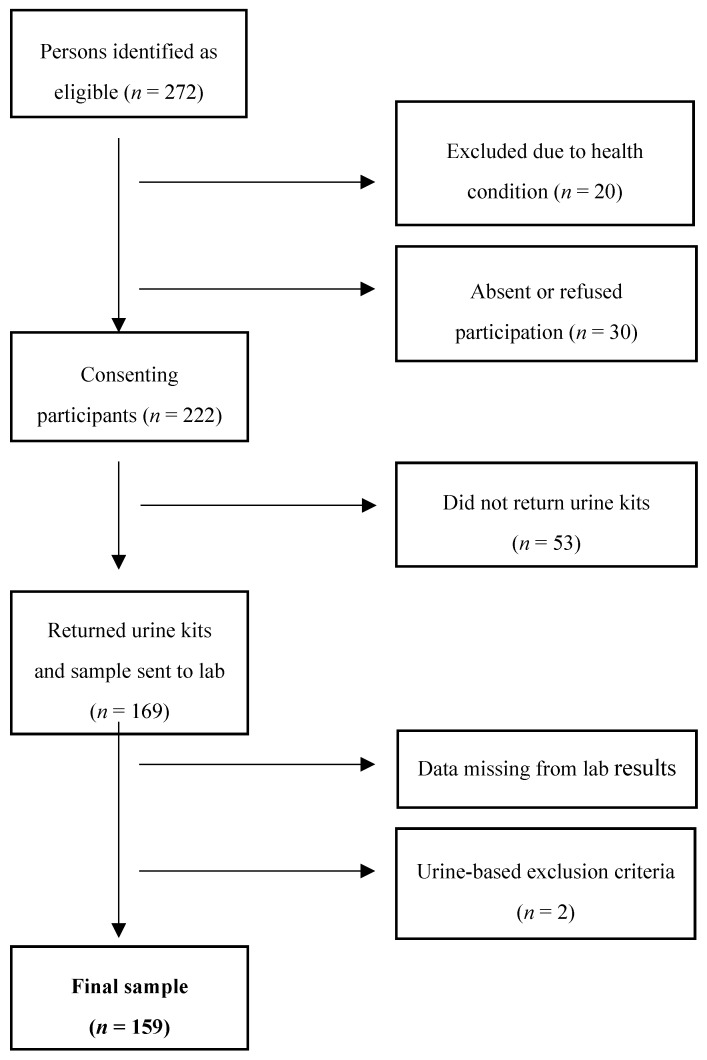
Flow chart illustrating formation of final sample population.

Figure 1 shows a flow chart of sample population. There were 272 persons identified as eligible, however 30 persons refused to participate. Participants were excluded if they were pregnant, affected by kidney failure, hemorrhage, liver disease, or had begun diuretic medication less than two weeks prior to recruitment (*n* = 20) [2]. A total of 222 participants were included in the study and invited to participate in urine collection. However, 53 participants did not return their urine collection kits after several attempts at follow-up. The number of urine samples sent to the laboratory was therefore 169. There were eight samples that were not analyzed for sodium and/or potassium and/or urine volume. The final study sample involved 161 participants (76 men and 86 women).

Two participants were not included in the analysis as they did not meet our criteria for complete 24-hour collection. This was assessed as 24-h creatinine <4 mmol/24 h for women and <6 mmol/24 h for men, or <500 mL urine collected/24 h [11]. One identified woman had creatinine of 2.18 mmol/24 h and one identified man had creatinine of 3.55 mmol/24 h—both just over half the minimum creatinine level required for inclusion with respect to their genders. Therefore, in total 159 cases were used to estimate 24-hour urinary electrolytic content and included in the analysis. One participant did not complete the questionnaire, but was still included in the analysis for SBP/DBP and urine excretion.

### 2.5. Ethics

The study was approved by the Regional Committee for Medical and Health Research Ethics (study code: 2015/1552 REK South-East). Informed written consent was obtained from all participants.

### 2.6. Statistical Analysis

Estimated daily sodium chloride intake (NaCl g/24 h) was calculated from sodium excretion using Formula [18]:

$$NaCl\;\left(g/24h\right)\;=Na\;\left(mmol/24h\right)\;\cdot \frac{58.4}{1000}\;$$

The molar ratio of sodium to potassium was also calculated: $\frac{Na\;\left(mmol/24h\right)}{K\;\left(mmol/24h\right)}$.

Mean and standard deviation (SD) were described for all continuous variables. Number and percentage were reported for categorical variables. Sodium intake is likely to differ by gender. Therefore results are shown separately for men and women. Differences between gender were tested with the independent samples *T*-test. The Mann-Whitney *U*-test was used to test the difference in data sets of non-parametric distribution.

For normally distributed variables, Pearson’s *r* was computed to assess correlation between demographic variables and 24-hour sodium excretion. Spearman’s *Rho* correlation coefficient was used to assess correlations for non-parametric distributions. This was similarly conducted to assess the correlation between 24-hour sodium excretion and both systolic blood pressure (SBP) and diastolic blood pressure (DBP).

Multiple linear regression—shown as β, 95% confidence interval (CI)—was used to examine the association between demographic variables (including gender, age, length of stay in Norway, and number of years of education) and 24-hour sodium excretion. The relation between 24-hour sodium excretion and SBP (also DBP) was similarly modelled using multiple linear regression, adjusting for confounding variables. Statistical assumptions were checked in the final models.

Collected data were described and analyzed using IBM SPSS Statistics, Version 24. Tests for differences were two-sided. The significance level was set to *p* < 0.05.

## 3. Results

Demographic characteristics and blood pressure data are described in Table 1. Mean age was 40.2 years. Men were, on average, older than women. However, men and women had spent approximately the same number of years living in Norway. Only two participants (1.2%) were born in Norway. Men were significantly more educated than women. SBP and DBP were significantly higher in men compared to women (*p* < 0.001 for SBP and *p =* 0.003 for DBP). A considerable proportion of participants were hypertensive (27.3%).

Table 2 shows levels of excreted sodium and potassium in men, women, and in total. Assuming all sodium from dietary intake was excreted through the urine, excretion corresponded to a 24-hour dietary salt intake of 8.66 g in men and 7.39 g in women. Potassium excretion was approximately 12 mmol/24 h higher in men compared to women. The Na:K ratio was 2.5 in men and 2.4 in women, and were not significantly different (Table 2).

In Table 3, multiple linear regression analysis between socio-demographic factors (gender, age, years lived in Norway, and years of education) and 24-hour sodium excretion demonstrated that excretion decreased by 1.5 mmol/24 h per year of age when adjusted by gender (*β* = −1.5; CI = −2.4, −0.7). Years lived in Norway and years of education showed no significant association with 24-hour sodium excretion (Table 3). Additionally, multiple linear regression showed that for each 100 mmol/24 h increase in sodium excretion, SBP increased by 1.2 mmHg (CI = −3.2, 5.6) and DBP decreased with 0.8 mmHg (CI = −3.2, 1.6). However, these associations were not significant.

## 4. Discussion

### 4.1. Summary of Findings

In this first study of salt intake among adult Somalis (age 20–67) in Norway, we estimated based on 24-hour urine collection that men consumed 8.66 g of salt and women consumed 7.39 g of salt per 24 h (or per day). In addition to male gender, younger age was found to be associated with increased salt intake. In the current study, estimated sodium intake was not significantly associated with SBP or DBP.

### 4.2. Context and Added Understanding

There have been few population-based studies measuring sodium intake among Norwegian residents. A previous study by Omvik et al. published in 1983 [19] reported markedly higher (28%) urinary sodium excretion in ethnic Norwegian men (192. 5 ± 76.4 mmol/24 h) compared to our results. A recent study conducted in 2015–2016 and so far published as an abstract, reported an estimated salt intake of 10.2 (±4.0) g/day in men and 7.4 (±2.7) g/day in women in an ethnic Norwegian population [20]. In comparison, men in our study population were estimated to consume substantially less salt, while women consumed similar amounts. The dietary sources of salt among these two populations are speculated to differ, with higher bread and processed food intake among ethnic Norwegians, possibly accounting for the difference seen in men. However, comparable data on the sources of dietary salt among Somalis in Norway does not exist.

Among Scandinavian populations, a study from Finland conducted in 2002 reported an estimated sodium excretion of 160.2 mmol/24 h in men and 124.7 mmol/24 h in women [21]. Compared to our results, Finnish men excreted slightly more sodium than Somali men in Norway. Differences between the women were negligible. Among a randomly selected group of young Swedish men, sodium excretion was reported as 198.0 ± 69 mmol/24 h—much higher compared to our results [22].

In a systematic review of 24-hour sodium excretion and dietary surveys in 51 studies from Western Europe, Powles et al. [23] found that estimated mean sodium intake ranged from 3.28 to 4.43 g/24 h, corresponding to a salt intake of 8.33 to 11.25 g/day. Our results for Somali adults are situated near the bottom of this range. Oyebode et al. [24] conducted a systematic review on sodium intakes in sub-Saharan Africa, finding all populations since 1990 consumed over 2 g/24 h, with the highest reliable intake estimates to be between 5 and 6 g/24 h in Tanzania, South Africa, and Ghana.

Only one study exists, to our knowledge, reporting 24-hour sodium excretion from a Somali population. Modesti et al. [25] reported sodium excretion of 97 mmol/24 h in 25 young Somali immigrants in Florence, Italy upon arrival and a significant increase to 165 mmol/24 h after six months of residence. This was accompanied by an 11 mmHg increase in SBP. Our results are similar to the reported sodium excretion after 6 months, suggesting Somalis in Oslo may have a similarly western-acculturated diet with respect to salt [25]. Health implications of this potential dietary change could apply to Somalis living in Norway, making it important to continue monitoring lifestyle risk factors, sources of dietary salt, and health outcomes [26,27].

Men were found to consume significantly more sodium than women, which is reported in the majority of previous studies investigated [8,23,28], with the exception of Oyebode et al.’s [24] systematic review of sodium intake in Sub-Saharan Africa. This is likely due to higher food consumption in men. This study also suggests an inverse relationship between salt intake and age. We hypothesize that this could be a result of increased consumption of fast-food as well as pre-prepared food among younger people, which tend to have higher salt contents than home-made meals.

A decreased Na:K ratio has emerged as an important factor conferring protection against hypertension and CVD. Potassium has consistently shown blood pressure-lowering effects by interacting with salt-sensitive processes to mitigate the blood pressure increasing effects of dietary sodium [1,29,30,31]. Fruits and vegetables are foods typically rich in potassium. Na:K ratios worldwide demonstrate a wide range, predominantly higher than the unofficial WHO recommendation of 1:1 [32]. Our results are reflective of this, with a large variance and values in the range of other reports. The highest reported ratio from Western European populations is, to our knowledge, the ratio of 3.23 found in the Gubbio population in Italy [1]. At the lower limit, Laatikainen et al. [21] reported ratios of 2.08 for men and 1.92 for women in the Finnish population. The Somali population in Florence from Modesti et al. [25] demonstrated increased sodium compared to potassium intake after six months of residence in Italy—from 2.02 (at arrival) to 3.0 (after six months). In comparison, our study population has an intermediate ratio. While our Na:K results are similar to previous studies, from an absolute perspective, our study population consumes less potassium compared to most populations, which suggests a lower fruit and vegetable intake.

There was no significant association between sodium intake and blood pressure (SBP and/or DBP) in our study, which is contrary to many comparable studies, [1,33,34]. One reason for this may be that there is individual day-to-day variability in the diet, which affects the given 24-hour sodium excretion. While 24-hour excretion is considered the best indicator for population intake, one single 24-hour excretion is not a precise indicator of a person’s habitual intake [12,35]. In addition, certain recent meta-analyses have demonstrated a small and/or weak relationship between sodium intake and blood pressure in populations consuming under 217.39 mmol/24 h of sodium and in normotensive populations [5,29]. Our sample has a relatively low prevalence of hypertension, with the majority of participants excreting less than 217.39 mmol/24 h of sodium.

### 4.3. Strengths

We approached this study with standardized methods using 24-hour urinary excretion (considered the gold standard) to estimate dietary intake of salt, sodium, and potassium alongside a validated protocol for collection/analysis as well as adherence to WHO’s recommended framework for NCD surveillance (STEPS) [17]. In addition, recruitment was community-based, focusing on Norway’s largest non-Western immigrant population, allowing us to gain understanding of their demographics, sodium and potassium intake, and blood pressure status—important background information for effectively improving the health status of marginalized populations [36,37,38].

### 4.4. Limitations

This study has some limitations. The exact number of Somalis in Sagene borough is not known due to frequent and often undocumented movement between boroughs in Oslo and out of the city. There were, however, an estimated 1200 Somalis from age zero registered in the borough in 2015. This population is estimated to be very young. Selection bias may be present as there were some eligible members of the Somali community in the borough who declined participation or could not be reached. In addition, this study selected participants from only one out of 15 districts in Oslo, which may not be representative of Somalis living in other parts of the country. On the other hand, 24-hour sodium excretion was not associated with length of education and how long they had been living in Norway, which both are important sociodemographic variables. We managed to reach 272 Somali adults in the borough (*n* = 272), collecting demographic data from 222, and urine from 159 individuals. Demographic characteristics were comparable between those who returned their urine kits and the 53 persons who did not.

Despite using gold standard methods for estimating salt intake, the collection of one sample per participant is a limitation. Conducting repeated collections and analyses was not feasible. Nevertheless, one-time collection of 24-hour urine is considered a highly valid method for estimating salt intake at the population level [10,12,13]. Still, our results should be interpreted with caution with respect to generalizability of the findings.

## 5. Conclusions

While above the WHO recommended intake for salt, the estimated salt intake in this Somali immigrant population is within the lower range of salt intakes globally and in Western Europe. Younger men were found to have the highest intake. This study is the first of its kind of an immigrant population in Norway, and it contributes to the small number of studies estimating salt intake in immigrant groups globally. It provides important baseline data on salt intake for comparison to other immigrant groups and ethnic Norwegians, and for inclusion in follow-up studies of longitudinal design.

## Figures and Tables

**Table 1:** Clinical and demographic characteristics of participants, according to gender. BP: blood pressure.

	Men (*n* = 74; 46.8%)		Women (*n* = 84; 53.2%)		Total (*n* = 158)
	*Mean (SD)*		*Mean (SD)*	*p* ^a^	*Mean (SD)*
Age (years)	42.3 (11.3)		38.4 (10.5)	0.026	40.3 (11.1)
Years lived in Norway	13.6 (7.5)		12.1 (6.1)	0.161	12.8 (6.8)
Years of education	11.9 (4.0)		8.0 (4.9)	<0.001	9.9 (4.9)
		*N (%)*	*N (%)*		*N (%)*
Marital status	*Married*	56 (75.7)	51 (60.7)		107 (67.7)
	*Single, separated or divorced*	18 (24.3)	33 (39.3)		51 (32.3)
Systolic BP (mmHg)	129.9 (16.9)		118.3 (18.0)	<0.001	123.8 (18.3)
Diastolic BP (mm Hg)	83.3 (10.0)		78.7 (8.6)	0.003	80.9 (9.6)

^a^*p* represents asymptotic significance.

**Table 2:** Urine analysis laboratory data of participants who returned complete 24-hour urine samples, according to gender.

	Men (*n* = 75)	Women (*n* = 84)		Total (*n* = 159)
	*Mean (SD)*	*Mean (SD)*	*p*	*Mean (SD)*
Urine volume (mL/24 h)	2061 (775)	1780 (649)	0.014	1913 (722)
Creatinine (mmol/24 h)	15.8 (3.9)	10.3 (4.8)	<0.001	12.9 (5.2)
Na (mmol/24 h)	150.6 (57.0)	126.6 (62.4)	0.013	137.9 (61.3)
K (mmol/24 h)	66.9 (25.5)	54.8 (19.5)	0.001	60.5 (23.3)
Na:K	2.5 (1.2)	2.4 (1.1)	0.665	2.4 (1.1)
NaCl (g/24 h)	8.66 (3.33)	7.39 (3.64)	0.013	8.05 (3.58)

**Table 3:** Multiple linear regression showing the relationship between daily sodium excretion (mmol/24 h) and demographic factors; univariate (crude) and adjusted estimates are shown.

	Crude β Estimate	95% CI		*p*	Adjusted β ^a^	95% CI		*p*
		Lower	Upper			Lower	Upper	
Years lived in Norway ^b^	−0.03	−1.41	1.47	0.97	0.2	−1.30	1.64	0.82
Years of education ^c^	2.6	0.6	4.5	0.01	0.1	−0.7	3.6	0.19

^a^ β coefficients for each demographic factor were adjusted for by inclusion of relevant confounders. ^b^ The factor *years lived in Norway* was adjusted by *gender*, *age*, and *years of education.* ^c^ The factor *years of education* was adjusted by *gender*, *age*, and *years lived in Norway.*

## References

[1] Intersalt Cooperative Research Group (1988). INTERSALT: An international study of electrolyte excretion and blood-pressure—Results for 24 hour urinary sodium and potassium excretion. Br. Med. J..

[2] Ha S.K. (2014). Dietary salt intake and hypertension. Electrol. Blood Press..

[3] He F.J., Li J., Macgregor G.A. (2013). Effect of longer-term modest salt reduction on blood pressure. Cochrane Database Syst. Rev..

[4] Mente A., O’Donnell M., Rangarajan S., Dagenais G., Lear S., McQueen M., Diaz R., Avezum A., Lopez-Jaramillo P., Lanas F. (2016). Associations of urinary sodium excretion with cardiovascular events in individuals with and without hypertension: A pooled analysis of data from four studies. Lancet.

[5] Graudal N.A., Hubeck-Graudal T., Jurgens G. (2017). Effects of low sodium diet versus high sodium diet on blood pressure, renin, aldosterone, catecholamines, cholesterol, and triglyceride. Cochrane Database Syst. Rev..

[6] GBD 2015 Risk Factors Collaborators (2016). Global, regional, and national comparative risk assessment of 79 behavioural, environmental and occupational, and metabolic risks or clusters of risks, 1990–2015: A systematic analysis for the Global Burden of Disease Study 2015. Lancet.

[7] GBD 2015 Mortality and Caues of Death Collaborators (2016). Global, regional, and national life expectancy, all-cause mortality, and cause-specific mortality for 249 causes of death, 1980–2015: A systematic analysis for the Global Burden of Disease Study 2015. Lancet.

[8] Brown I.J., Tzoulaki I., Candeias V., Elliott P. (2009). Salt intakes around the world: Implications for public health. Int. J. Epidemiol..

[9] World Health Organization (2012). Guideline: Sodium Intake for Adults and Children.

[10] McLean R.M. (2014). Measuring population sodium intake: A review of methods. Nutrients.

[11] Land M.-A., Webster J., Christoforou A., Praveen D., Jeffery P., Chalmers J., Smith W., Woodward M., Barzi F., Nowson C. (2014). Salt intake assessed by 24 h urinary sodium excretion in a random and opportunistic sample in Australia. BMJ Open.

[12] Hunter D., Van Dam R., Willett W. (2013). Biochemical Indicators of Dietary Intake. Nutritional Epidemiology.

[13] Wielgosz A., Robinson C., Mao Y., Jiang Y., Campbell N.R., Muthuri S., Morrison H. (2016). The Impact of Using Different Methods to Assess Completeness of 24-Hour Urine Collection on Estimating Dietary Sodium. J. Clin. Hypertens. (Greenwich).

[14] Kumar G.L., Meyer H.E., Søgaard A.J., Strand B.H. (2008). The Oslo Immigrant Health Profile.

[15] Roche Diagnostics (2005). 510(k) Summary—ISE Indirect Na, K, CI for Gen.2.

[16] Cao X., Song C., Guo L., Yang J., Deng S., Xu Y., Chen X., Sapa W.B., Wang K. (2015). Quality Control and Validation of Oscillometric Blood Pressure Measurements Taken During an Epidemiological Investigation. Medicine.

[17] World Health Organization (2003). The WHO STEPwise Approach to Surveillance of Noncommunicable Diseases (STEPS).

[18] Huang L., Crino M., Wu J.H.Y., Woodward M., Barzi F., Land M.-A., McLean R., Webster J., Enkhtungalag B., Neal B. (2016). Mean population salt intake estimated from 24-h urine samples and spot urine samples: A systematic review and meta-analysis. Int. J. Epidemiol..

[19] Omvik P., Lund-Johansen P., Eide R. (1983). Sodium excretion and blood pressure in middle-aged men in the Sogn County: An intra- and interpopulation study. J. Hypertens..

[20] Meyer H.E., Johansson L., Eggen A., Holvik K. (2017). Salt intake assessed by 24-hour urine excretion in the Tromso Study 2015–2016. Eur. J. Prev. Cardiol..

[21] Laatikainen T., Pietinen P., Valsta L., Sundvall J., Reinivuo H., Tuomilehto J. (2006). Sodium in the Finnish diet: 20-year trends in urinary sodium excretion among the adult population. Eur. J. Clin. Nutr..

[22] Hulthen L., Aurell M., Klingberg S., Hallenberg E., Lorentzon M., Ohlsson C. (2010). Salt intake in young Swedish men. Public Health Nutr..

[23] Powles J., Fahimi S., Micha R., Khatibzadeh S., Shi P., Ezzati M., Engell R.E., Lim S.S., Danaei G., Mozaffarian D. (2013). Global, regional and national sodium intakes in 1990 and 2010: A systematic analysis of 24 h urinary sodium excretion and dietary surveys worldwide. BMJ Open.

[24] Oyebode O., Oti S., Chen Y.F., Lilford R.J. (2016). Salt intakes in sub-Saharan Africa: A systematic review and meta-regression. Popul. Health Metr..

[25] Modesti P.A., Tamburini C., Hagi M.I., Cecioni I., Migliorini A., Neri Serneri G.G. (1995). Twenty-four-hour blood pressure changes in young Somalian blacks after migration to Italy. Am. J. Hypertens..

[26] Sanou D., O’Reilly E., Ngnie-Teta I., Batal M., Mondain N., Andrew C., Newbold B.K., Bourgeault I.L. (2014). Acculturation and nutritional health of immigrants in Canada: A scoping review. J. Immigr. Minor. Health.

[27] Venters H., Gany F. (2011). African Immigrant Health. J. Immigr. Minor. Health.

[28] Land M.A., Neal B.C., Johnson C., Nowson C.A., Margerison C., Petersen K.S. (2018). Salt consumption by Australian adults: A systematic review and meta-analysis. Med. J. Aust..

[29] Mente A., O’Donnell M.J., Rangarajan S., McQueen M.J., Poirier P., Wielgosz A., Morrison H., Li W., Wang X., Di C. (2014). Association of Urinary Sodium and Potassium Excretion with Blood Pressure. N. Engl. J. Med..

[30] Pilic L., Pedlar C.R., Mavrommatis Y. (2016). Salt-sensitive hypertension: Mechanisms and effects of dietary and other lifestyle factors. Nutr. Rev..

[31] O’Donnell M., Mente A., Rangarajan S., McQueen M.J., Wang X.Y., Liu L.S., Yan H., Lee S.F., Mony P., Devanath A. (2014). Urinary Sodium and Potassium Excretion, Mortality, and Cardiovascular Events. N. Engl. J. Med..

[32] World Health Organization (2012). Guideline: Potassium Intake for Adults and Children.

[33] Aburto N.J., Ziolkovska A., Hooper L., Elliott P., Cappuccio F.P., Meerpohl J.J. (2013). Effect of lower sodium intake on health: Systematic review and meta-analyses. BMJ.

[34] Stamler J., Chan Q., Daviglus M.L., Dyer A.R., Van Horn L., Garside D.B., Miura K., Wu Y., Ueshima H., Zhao L. (2018). Relation of Dietary Sodium (Salt) to Blood Pressure and Its Possible Modulation by Other Dietary Factors: The INTERMAP Study. Hypertension.

[35] Olde Engberink R.H.G., van den Hoek T.C., van Noordenne N.D., van den Born B.H., Peters-Sengers H., Vogt L. (2017). Use of a Single Baseline Versus Multiyear 24-Hour Urine Collection for Estimation of Long-Term Sodium Intake and Associated Cardiovascular and Renal Risk. Circulation.

[36] Gele A.A., Pettersen K.S., Kumar B., Torheim L.E. (2016). Diabetes Risk by Length of Residence among Somali Women in Oslo Area. J. Diabetes Res..

[37] Kumar R., Einstein G. (2012). Cardiovascular disease in Somali Women in the Diaspora. Curr. Cardiovas. Risk Rep..

[38] Kinzie J.D., Riley C., McFarland B., Hayes M., Boehnlein J., Leung P., Adams G. (2008). High prevalence rates of diabetes and hypertension among refugee psychiatric patients. J. Nerv. Ment. Dis..

